# Further Theoretical Insight into the Mechanical Properties of Polycaprolactone Loaded with Organic–Inorganic Hybrid Fillers

**DOI:** 10.3390/ma11020312

**Published:** 2018-02-21

**Authors:** Saverio Maietta, Teresa Russo, Roberto De Santis, Dante Ronca, Filomena Riccardi, Michelina Catauro, Massimo Martorelli, Antonio Gloria

**Affiliations:** 1 Department of Industrial Engineering, Fraunhofer JL IDEAS–University of Naples Federico II, P.le Tecchio 80, 80125 Naples, Italy; smaietta@unina.it (S.M.); massimo.martorelli@unina.it (M.M.); 2 Institute of Polymers, Composites and Biomaterials—National Research Council of Italy, V.le J.F. Kennedy 54–Mostra d’Oltremare Pad. 20, 80125 Naples, Italy; teresa.russo@unina.it (T.R.); rosantis@unina.it (R.D.S.); 3 Institute of Orthopaedics and Traumathology, University of Campania “Luigi Vanvitelli”, Via L. De Crecchio, 2-4, 80138 Naples, Italy; dante.ronca@unicampania.it (D.R.); mena.riccardi@libero.it (F.R.); 4 Department of Industrial and Information Engineering, University of Campania “Luigi Vanvitelli”, Via Roma 29, 81031 Aversa, Italy; michelina.catauro@unicampania.it

**Keywords:** computer-aided design (CAD), mechanical analysis, finite element analysis (FEA), composites, organic–inorganic hybrid materials, biomedical applications

## Abstract

Experimental/theoretical analyses have already been performed on poly(ε-caprolactone) (PCL) loaded with organic–inorganic fillers (PCL/TiO_2_ and PCL/ZrO_2_) to find a correlation between the results from the small punch test and Young’s modulus of the materials. PCL loaded with Ti2 (PCL = 12, TiO_2_ = 88 wt %) and Zr2 (PCL = 12, ZrO_2_ = 88 wt %) hybrid fillers showed better performances than those obtained for the other particle composition. In this context, the aim of current research is to provide further insight into the mechanical properties of PCL loaded with sol–gel-synthesized organic–inorganic hybrid fillers for bone tissue engineering. For this reason, theoretical analyses were performed by the finite element method. The results from the small punch test and Young’s modulus of the materials were newly correlated. The obtained values of Young’s modulus (193 MPa for PCL, 378 MPa for PCL/Ti2 and 415 MPa for PCL/Zr2) were higher than those obtained from a previous theoretical modelling (144 MPa for PCL, 282 MPa for PCL/Ti2 and 310 MPa for PCL/Zr2). This correlation will be an important step for the evaluation of Young’s modulus, starting from the small punch test data.

## 1. Introduction

In the field of tissue engineering, the development of advanced substrates and scaffolds represents a great challenge. As reported in the literature, different kinds of polymers and polymer-based composite materials have been widely proposed together with several approaches to improve their biological and mechanical features [1,2,3,4,5,6].

Organic–inorganic hybrid materials have been synthesized and proposed as biomaterials with interesting properties [7,8,9,10,11,12,13,14,15].

Most hybrid organic–inorganic materials can be synthesized via the sol–gel method, benefiting from the combination of the best characteristics of polymers and inorganic materials.

The sol–gel chemistry is based on the hydrolysis and polycondensation of metal alkoxides (M(OR)*_x_* with M = Si, Sn, Zr, Ti, Al, Mo, V, W, Ce, and so forth [1,7,8,16,17]). The great advantage of the sol–gel method results in a process which can be performed at low temperature (i.e., room temperature) [1,4,17].

Hybrid materials with different physical properties and morphologies have been developed, introducing many polymers (the organic phase) into inorganic networks [1,7]. Regarding synthetic polymers, poly(ε-caprolactone) (PCL), which is a biodegradable aliphatic polyester [4,18,19], has been widely investigated for several biomedical applications (i.e., tissue engineering) [5,6,20,21,22,23,24,25].

On the other hand, the bioactivity of ZrO_2_ and TiO_2_ glasses was previously demonstrated, highlighting the formation of a bonelike apatite layer on the surfaces, hence the ability to bond to living bone [1].

In addition, previous works focused on the synthesis of PCL-based organic–inorganic materials via the sol–gel method, where PCL was incorporated into the network by means of hydrogen bonds between the carboxylic groups of the polymer and the hydroxyl groups of the inorganic phase, as well as on the analysis of the developed PCL/TiO_2_ and PCL/ZrO_2_.

The formation of a hydroxyapatite layer on the surfaces of samples soaked in a solution with a composition that simulated human blood plasma demonstrated the bioactivity of PCL/ZrO_2_, PCL/TiO_2_, and further organic–inorganic hybrid materials [4,26,27].

With regard to the design of substrates for hard-tissue engineering, mechanical performances play an important role, as hard tissues (i.e., bone) are stronger (higher strength) and stiffer (higher elastic modulus) compared to soft tissues (i.e., cartilage) [1,4].

Generally, polymers such as PCL do not match the required mechanical properties, as they are too flexible and weak, if hard-tissue engineering applications are considered. For this reason, to overcome these drawbacks, an alternative choice is represented by the use of polymer-based composites [1,2,3,4,5,6]. Benefiting from technical criteria and considerations in the design of composite materials, as well as from the concepts of the stress transfer mechanism and stress concentration, the amount of micro/nano-particles embedded in a polymer matrix may be properly optimized to avoid weakness in the structure [1,2,3,4,6]. The possibility to improve the properties of the neat PCL by embedding the bioactive PCL/TiO_2_ or PCL/ZrO_2_ organic–inorganic hybrid microfillers has already been demonstrated [1]. Specifically, it was found that both small punch tests and cell viability/proliferation analyses showed mechanical and biological performances for PCL reinforced with Ti2 (PCL = 12, TiO_2_ = 88 wt %) and Zr2 (PCL = 12, ZrO_2_ = 88 wt %) hybrid fillers, which were better than those obtained for the other particle composition [1].

In this context, starting from the optimization of 2D substrates, 3D additive manufactured composite scaffolds for hard-tissue engineering were developed and analyzed [4].

It is well known that the small punch test represents an interesting test method to evaluate the mechanical properties when a material is available only in small quantities [1,4]. However, this test method cannot be used for the determination of Young’s modulus. A numerical simulation is also needed [4].

Thus, finite element (FE) analysis was performed on 2D substrates consisting of PCL loaded with sol–gel-synthesized PCL/TiO_2_ or PCL/ZrO_2_ hybrid fillers [4]. The results from the small punch test and Young’s modulus of the materials were correlated [4].

As reported previously [4], the problem is axisymmetric and the model was previously meshed with a quadrilateral planar element in a representative axial section. In particular, six disk specimens with different values of Young’s modulus (200 MPa, 500 MPa, 1000 MPa, 2000 MPa, 3500 MPa, and 5000 MPa) were already simulated, assuming a Poisson’s ratio of 0.40. In any case, the obtained values were lower than the experimental Young’s modulus.

Taking into account the results from previous experimental tests [1], as well as the approach already reported for the FE analysis [4], in the current research, further theoretical analyses were carried out on the 2D substrates. This consisted of a PCL matrix loaded with sol–gel-synthesized PCL/TiO_2_ or PCL/ZrO_2_ hybrid fillers, with the aim of finding a new correlation between the small punch test data and Young’s modulus.

## 2. Results and Discussion 

The starting point of the current research was a theoretical approach reported in the literature [4], which benefited from experimental results on PCL loaded with sol–gel-synthesized PCL/TiO_2_ or PCL/ZrO_2_ particles [1]. Specifically, the present study reports theoretical analyses on the 2D substrates consisting of a PCL matrix loaded with sol–gel-synthesized PCL/Ti2 or PCL/Zr2 hybrid fillers (PCL = 12 and TiO_2_ = 88 wt % for Ti2; PCL = 12 and ZrO_2_ = 88 wt % for Zr2), to provide further insight into the mechanical properties. All the results were compared to those obtained from a previous experimental/theoretical approach [4].

The important role of CAD, image and theoretical/experimental analyses has been widely reported for different kinds of applications [28,29,30,31,32]. 

With regard to experimental analysis, the small punch test is considered as a reproducible miniature specimen test method to evaluate the mechanical properties of ultra-high molecular weight polyethylene used in surgical implants and retrieved acrylic bone cements [1,4]. However, it was also employed to assess the punching properties of PCL/iron-doped hydroxyapatite nanocomposite substrates and PCL loaded with sol–gel-synthesized organic–inorganic hybrid fillers [1,2].

Previous experimental data from small punch tests on PCL and PCL loaded with sol–gel-synthesized organic–inorganic hybrid fillers showed load-displacement curves generally displaying an initial linear trend, a successive decrease in the curve slope until a maximum load was reached, and a final decrease in the load values until failure occurred. Furthermore, among the investigated composite substrate, better mechanical and biological features were found for PCL reinforced with Ti2 (PCL b = 12, TiO_2_ = 8 wt %) and Zr2 (PCL = 12, ZrO_2_ = 8 wt %) hybrid fillers [1,4].

In the present FE analysis, similar results were clearly achieved in terms of the displacement contour plot for all composites when different loads were applied according to Young’s modulus of each disk specimen (from 200 to 5000 MPa) to obtain a final displacement value of 0.2 mm.

**Figure 1:**
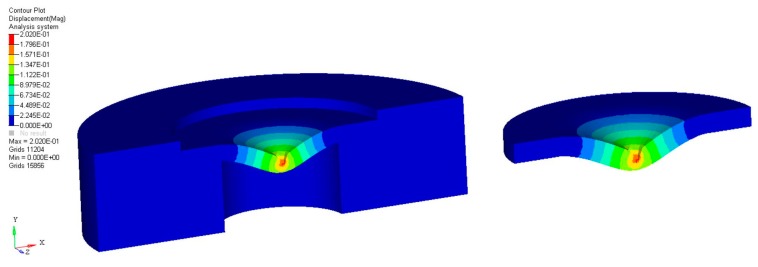
Results from finite element (FE) analysis: typical displacement contour plot for a composite disk.

As an example, a displacement contour plot for a composite disk is shown in Figure 1.

Basically, the area of interest is represented by the disk placed in/on the support. As expected, the maximum displacement values (0.2 mm) were achieved in the center zone (red color) of the specimen.

**Figure 2:**
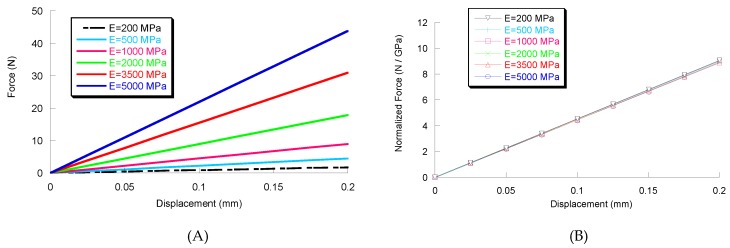
Results from FE analysis: force–displacement curves (**A**) and normalized force–displacement curves (**B**).

Accordingly, FE analysis was used to calculate the force–displacement curves (Figure 2A) for all of the investigated materials, as well as a normalized force–displacement curve (Figure 2B) which was obtained by dividing the force value by Young’s modulus for all materials.

Even though the problem was non-linear as a consequence of the friction, it was found that all the points of the normalized force–displacement curves collapsed into a single curve (Figure 2B).

The single curve was approximated according to the following equation:

(1) $$\frac{F}{E}=A\cdot \delta$$

where *F* is the applied force, *E* represents Young’s modulus of the material, *A* is a constant equal to 45.1 N·GPa^−1^·mm^−1^, and *δ* is the displacement of the punch.

Computational results and Equation (1) may represent interesting findings, providing the possibility to assess Young’s modulus of materials subjected to the small punch test, once the value of the Poisson’s ratio (close to 0.40) and the coefficient of friction (around 0.20) are considered.

Thus, according to Equation (1), the values of Young’s modulus (which better describe the experimental results from small punch tests performed on PCL, PCL/Ti2 and PCL/Zr2 [1,4]) are shown in Table 1.

Even though Young’s modulus evaluated for PCL (193 MPa) was higher than that already assessed by a previous theoretical analysis (144 MPa) [4], it is still lower if compared to the neat PCL (i.e., 343.9–571.5 MPa) [4,33,34]. Such lower value should probably be ascribed to the intrinsic porosity of the substrate as a direct consequence of the solvent evaporation during the preparation via moulding and solvent casting techniques [1,4].

In addition, Young’s modulus evaluated for PCL/Ti2 (378 MPa) and PCL/Zr2 (415 MPa) was also higher than those previously computed in theoretical modelling (282 MPa and 310 MPa) [4].

The higher values of Young’s modulus obtained for PCL, PCL/Ti2 and PCL/Zr2 would seem to suggest a better approximation of the experimental values.

In a previous work on advanced composites for hard-tissue engineering (based on PCL/organic–inorganic hybrid fillers to design 3D additive manufactured scaffolds starting from 2D substrates [4]), results from compression tests on 3D porous structures showed mean values of compressive modulus which were 90 MPa and 79 MPa for 3D composite (PCL/Ti2, PCL/Zr2) and PCL scaffolds, respectively. Such values were lower than Young’s moduli obtained in the current research (Table 1) and those computed in the previous theoretical modelling [4], as a consequence of the controlled morphology and macro-porosity of the 3D additive manufactured scaffolds, where “apparent” stress and strain were considered.

A potential limitation of the current study was the lack of correlation between the obtained results and the preparation method (i.e., moulding and solvent casting techniques), which clearly affects the morphology, surface topography, and, consequently, the punching performances. In any case, the present work should be considered as further insight into the mechanical properties of the analyzed microcomposites, as well as an initial step towards future research with the aim of finding a complex correlation, which will also involve the effect of the preparation method.

## 3. Materials and Methods

### 3.1. Materials

Class I PCL/TiO_2_ and PCL/ZrO_2_ organic–inorganic hybrid particles were synthesized via the sol–gel method, and composites consisting of poly(ε-caprolactone) (PCL) loaded with the hybrid particles were developed, as described elsewhere [1]. Briefly, in designing PCL-based composite substrates, PCL pellets (weight-average molecular weight M_w_ = 65,000, Aldrich, St. Louis, MO, USA) were dissolved in tetrahydrofuran (THF, Aldrich, St. Louis, MO, USA) with stirring at room temperature [1].

Organic–inorganic particles with a diameter lower than 38 µm were dispersed in the polymer solution (ultrasonic bath, Branson 1510 MT, Danbury, CT, USA). Moulding and solvent casting techniques were used to manufacture disk-shaped specimens (6.4 mm in diameter and 0.5 mm in thickness) [1,2].

### 3.2. Generation of Solid Model and Numerical Simulation

The 3D CAD (computer-aided design) model was obtained using SolidWorks^®^ software (2016, Waltham, MA, USA). All the elements were considered as volumes and then connected. The assembly function was suitably employed and the model was built up.

**Figure 3:**
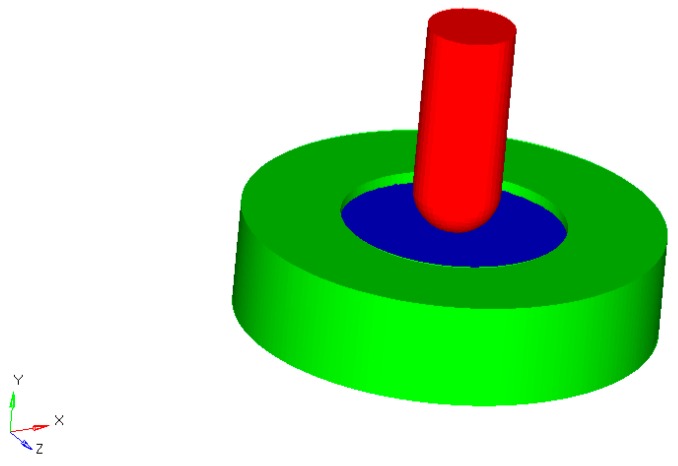
Schematic representation of the geometric model.

The geometric model related to the small punch test is schematically reported in Figure 3. The disk specimen is pictured in blue, whereas the steel punch and support are in red and green, respectively.

The geometric model of the small punch test was imported into the HyperWork^®^ 14.0 (Altair Engineering Inc., Troy, MI, USA) environment using the STEP (Standard for the Exchange of Product) format, and the finite element analysis (FEA) was performed. In the model, all the materials were assumed to be linearly elastic and isotropic. Each component of the model was defined in terms of mechanical properties (i.e., Young’s modulus and Poisson’s ratio).

The values of Young’s modulus and Poisson’s ratio considered for the different materials are listed in Table 2. In particular, according to a previous work [4], with regard to the steel, a modulus of 210 GPa and a Poisson’s ratio of 0.35 were assumed, and six disk specimens with different values of Young’s modulus (200 MPa, 500 MPa, 1000 MPa, 2000 MPa, 3500 MPa, and 5000 MPa) were simulated. In addition, a Poisson’s ratio of 0.40 was assumed for all the disk specimens [4]. Contact elements were used to model the contact between disk and punch as well as between disk and support, assuming a friction coefficient of 0.20 for all the mating surfaces [4].

In a previous study [4], the geometry of the test was reproduced using the commercial finite element software ANSYS 11 (Ansys Inc., Canonsburg, PA, USA) and the model was meshed only with quadrilateral planar element (PLANE82) in a representative axial section (as the problem is axisymmetric). In the present research, a 3D mesh was created and each model was divided into 3D four-sided solid elements (CTETRA). With the aim to minimize the mesh-dependent results, an appropriate mesh size was employed for all components of the model.

Table 3 shows the number of grids, elements, contact elements, and degrees of freedom for the analyzed model.

The results previously obtained from experimental analysis (small punch test-ASTM F2183) [1] and theoretical modelling [4] on 2D disk specimens (6.4 mm in diameter and 0.5 mm in thickness), consisting of PCL loaded with sol–gel-synthesized organic–inorganic hybrid fillers, were also considered.

## 4. Conclusions

The following conclusions were drawn:

1. The results from the small punch test and Young’s modulus of PCL loaded with sol–gel-synthesized organic–inorganic hybrid fillers were newly correlated.
2. The data were compared to those obtained from a previous theoretical modelling, suggesting a better approximation of the experimental Young’s modulus.
3. The found correlation will be an important step in the evaluation of Young’s modulus starting from the small punch test data.

## Figures and Tables

**Table 1:** Results obtained from theoretical analysis (Young’s modulus) benefiting from experimental small punch tests (displacement and force). The experimental results were adapted from [1,4].

Materials	Displacement (mm)	Force (N)	Young’s Modulus (MPa)
PCL	0.195	1.70	193
PCL/Ti2	0.191	3.26	378
PCL/Zr2	0.192	3.59	415

**Table 2:** Mechanical properties of materials: Young’s modulus and Poisson’s ratio. All values were adapted from [4].

Material	Young’s Modulus (MPa)	Poisson’s Ratio
Disk 1	200	0.40
Disk 2	500	0.40
Disk 3	1000	0.40
Disk 4	2000	0.40
Disk 5	3500	0.40
Disk 6	5000	0.40

**Table 3:** Number of grids, elements, contact elements and degrees of freedom for the analyzed model.

Total # of Grids	Total # of Elements	Total # of Contact Elements	Total # of Degrees of Freedom
55,559	267,342	1461	158,939
